# Mr. Howship on Diseases of the Lower Intestines and Anus, &c. &c. &c.

**Published:** 1820-09-01

**Authors:** 


*Practical Observations on the Symptoms, Discrimination, and Treatment, of some of the most common Diseases of the Lower Intestines, and Anus; particularly including those Affections produced by Stricture, Ulceration, and Tumour, within the Cavity of the Rectum; and Piles, Fistulæ, and Excrescences, formed at its External Opening. Illustrated by Cases. To which are added, some Suggestions upon a new and successful Mode of Correcting Habitual Confinement in the Bowels, to insure their regular Action without the Aid of Purgatives; on a Principle essentially conducive to the Prevention of the above Diseases.* By JOHN HOWSHIP, Member of the Royal College of Surgeons, in London. 1 vol. Octavo, pp. 176. London, 1820.

THE number of books which have lately issued from the press, on diseases of the pelvic viscera and lower intestines, would almost induce us to believe that the late epidemic had, to use the expression of Sydenham, "turned in upon the guts," in order to give the *surgeons* a share of the practice resulting from its wide-spreading influence. The establishment of dispensaries is a measure that must tend to give an amazing impulse to the progress of medical and surgical science. In these institutions diseases are seen *earlier* among the poor than they otherwise would; and as the medical officers, in general, are young, zealous, and not distracted by the multifarious exertions of extensive private practice, they study the phenomena of diseases more carefully, record them more accurately, and communicate them more freely, than before these eleemosynary mansions were erected. These advantages are certainly not without some alloy, as it respects medical *literature.* The just balance between facts and deductions; that is, between cases and principles, is somewhat deranged, and the press pours forth the *former* in such a torrent as threatens a deluge rather than a fertilizing irrigation. Nevertheless, as individual cases form the basis of medical science, in the same manner as grains of sand form the mountain, and drops of water the ocean, we must not object to this multiplication, in this infantile period of our knowledge.

Mr. Howship is favourably known to the profession by several preceding productions, both of his pen and pencil; and the opportunities for observation which he enjoys in public and private practice, together with his access to the valuable museum and manuscripts of his patron, Mr. Heaviside, afford him ample materials whereon to exercise the talent with which he is entrusted.

The first chapter of the work before us is on Stricture of the Rectum; but as Mr. Howship's observations do not differ essentially from what is stated by other writers, we shall pass over this chapter, with the exception of some remarks on tobacco injection, which will be found in our first article, on peritonitis.

The second chapter is on ulceration of the internal surface of the intestines. Although observation has proved that inflammation generally precedes ulceration in all structures of the body, yet our author does not think that this is *always* the case. He has detected absorption in the bones, unconnected with any character of preceding inflammation.

"In the dissection of those who have died from disease in the alimentary canal, I have, in various instances, (says Mr. H.) found so little trace of inflammatory action around spots of apparently recent ulceration, that I cannot help believing that, under some circumstances, irritation arising in the bowels, may establish a degree of excitement sufficient to induce ulceration, without any distinct sign of inflammatory action." 48.

We confess ourselves to be sceptical on this point. The appearances, *post mortem,* round an ulcerated spot, are not to be depended upon, especially in the mucous membranes, where all traces of prior inflammation may, and do often disappear after death, particularly where there is ulceration. And in a practical point of view, the inflammatory doctrine is the safest to bear in mind.

"In considering the occasional causes of irritation in the bowels, it has often appeared to me that the functions of the liver, and consequently the properties of the bile, are very much influenced by external circumstances; and that those who are but little exposed to the inclemency of weather, are nevertheless liable to suffer from an acrimony in the bilious secretion, as a consequence of common cold, an effect quite distinct from the increased quantity of thin mucous fluid excreted from the bowels in dysenteric diarrhœa; the first exciting a distressing sense of heat, and even excoriation about the anus; the second passing off without any such irritation, although they are both occasionally attended with an irksome sense of weight, and bearing down in the rectum." 48.

Ulceration, in its commencement, is generally attended with pain in some part of the abdominal region, usually acute, and more or less intense, according as it is of a phlegmonous or erysipelatous nature.

"Obstinate costiveness, extreme tenderness or severe pain in the belly, heat of skin, thirst, and white tongue, hard and quick pulse, will sometimes lead to a suspicion of acute inflammation, requiring diligent attention, and the most active treatment; while, in other cases, with heat of skin, thirst, foul tongue, and local pain, the pulse, although quickened, will not be remarkably hard." 49.

When ulceration follows as a consequence of the above, it will be either circumscribed or diffused. The former is the most dangerous, as the ulcer is more likely to penetrate through all the coats of the intestine, and thus prove mortal.

"In some cases, inflammation affects all the coats of the bowel at the same time, a circumstance that frequently is the means of saving the life of the patient. Coagulable lymph is poured out upon the bowel, producing adhesion either to the external parietes of the abdomen, or to some other part of the intestinal tube, by which assistance the ulcerative action making its way through the mass of lymph, produces an outlet for the contained matters through the external integuments, or effects a passage out of one into another part of the intestinal canal; in either case, preventing the mischief that would arise from the contents of the bowels escaping into the general cavity of the belly. Sometimes the adhesive process puts an entire stop to the further progress of mischief." 51.

Superficial ulcerations, our author thinks, may heal, and all their febrile accompaniments vanish. Mr. H. has, in several instances, visited persons attacked with severe pains in the bowels, attended with discharges of blood downwards and upwards. The attacks were continued some time, the fluids passed resembling thick, dark, bilious stools; at others, appearing like dark grumous blood. In these complaints, the fits of griping pain have occurred after the manner of spasm, being presently succeeded by a free evacuation, from which the patient has experienced temporary relief. In one case, which terminated fatally, Mr. Howship had an opportunity of examining the body.

"The bleeding had taken place from the capillary or exhalent arteries upon the internal surface of the great intestine, and although it was evident that every part of the bowel had been a bleeding surface, no part had suffered ulceration, nor was any portion inflamed, though the whole was very red." 54.

Mr. H. attributes this complaint to a scorbutic diathesis, although there was no attendant spongy state of the gums. It *is not*, indeed, easy to account for those sudden and local injections of the capillary vessels of certain tissues, but we see them in the membranes of the brain, in the vessels of the lungs, and of other parts; and they have been not inaptly termed *apoplexies* by the French pathologists.

In respect to the treatment of inflammation of the bowels, we shall only select the following passage, which will be found to bear on the discussion which we entered into respecting purgatives in Enteritis, in our leading article.

"A very essential, if not the most important point, consists in establishing a free and relaxed state of the bowels. Till this point is achieved, the patient cannot be considered safe; but this once effected, and febrile action somewhat relieved, the case will, or at least ought, to end well, with the assistance of proper saline or antimonial diaphoretics, and due attention to diet.." 59.

When ulceration has actually taken place in the mucous membrane of the bowels, the greatest attention will be necessary in respect to diet, in order to prevent the formation of acrimonious secretions in the canal. Mild diaphoretics, light tonics, aromatics, opiates, and castor oil, are the principal medicines to be used.

The 3d, 4th, 5th, 6th, and 7th chapters of the work, on Tumours of the Rectum, Prolapsus Ani, Hæmorrhoids, Fistula in Ano, and Hæmorrhoidal Excrescences, we must pass over, partly because they are not adapted for analysis, and partly because the subjects are already treated of in other parts of this Journal.

The eighth, or last chapter in the work, treats of the means best calculated to establish a regular state and action of the bowels: and here there is certainly some novelty. The circumstance that led Mr. Howship to adopt a new plan of treatment in habitual constipation of the bowels, was as follows:

"It happened that an elderly lady, residing at Scarborough, desired my opinion requesting me to consider of some plan, by the adoption of which she might obtain a more regular action of her bowels. She had no complaint to make as to her general health; her appetite was good, and she slept well; neither did there appear to be any material defect in the condition of the digestive organs, the only objectionable circumstance being that of her scarcely ever passing a stool without the assistance of medicine. The advice, she said, she had always received from her professional friends was, that, when confined in her bowels, she must still have recourse to opening medicines; she added, that really she had taken so great a variety, and so large a quantity, that she loathed the very idea of going on, and felt extremely anxious to know if any plan could be suggested to render it unnecessary.

"On reflection, it appeared probable, that this was an instance of deficient action from defective strength, and that, perhaps, by persevering for a time in the use of medicines calculated to restore tone, the bowels might recover the disposition, as well as the power, to propel their contents with regularity; at any rate, there could be no harm in making the experiment. I therefore first ordered the decoction and tincture of bark to be taken daily. This, in a week, appeared to have done neither good nor harm; there was no heat of tongue or skin; but there had been occasion for castor oil. Decoction of bark was next directed by itself; and in three weeks she thought her inside felt stronger, with less disposition to flatulence than before. In consequence of this amendment, the medicine was continued for a month longer; within which period, she found there was no longer any occasion to solicit the action of the bowels at all, a regular and easy motion occuring every day. This restoration in the tone and action of the bowels appeared likely to be lasting; for there had been no return of the complaint a year and a half afterwards." 163.

The adoption of a similar principle, with some slight modifications, our author informs us, has, in a variety of instances, enabled him to restore to the bowels the power of acting from their own impulse, without the perpetual necessity of purgatives. In these cases, Mr. H. at first combined the decoction of bark, with a fourth part of infusion of senna, or such proportion as answered the purpose of regulating the bowels, occasionally diminishing the quantity of the aperient, till the action of the bowels was observed to go on well with the bark alone.

We have no doubt that there are many cases in which the foregoing plan would be proper and efficacious; but, from considerable experience in this class of complaints, we are disposed to think that it would not be found generally applicable. In the majority of cases of constipation of the bowels, the cause will appear to be a deficient secretion in the mucous membrane of the intestines, and of the glandular organs, whose excretory ducts open therein, particularly the liver; and experience has proved, that the most effectual methodus medendi consists of such means as increase these secretions. This is the way in which purgatives themselves act; but every stimulus gradually loses its power by exhausting the excitability of the parts; and consequently requires corresponding augmentation of dose, as all people know, who are obliged to obviate constipation of the bowels by purgatives.

The *radical* treatment, therefore, should be founded on more general principles. We consider *exercise* as one of the greatest promoters of the internal, as well as the cutaneous secretions. If this measure alone were carried to a certain extent, it would remove constipation of the bowels in nine cases out of ten; but in the higher walks of life, and among the sedentary classes of all ranks, this measure can rarely be put in force.

The next most general remedy is to be applied through the medium of the skin. A critic, in a cotemporary Journal, has lately discovered a *reverse* sympathy between the skin and the glandular organs of the abdomen, and teaches us that when the functions of the skin are increased, those of the liver and intestines are lessened; and *vice versa*. Now this doctrine, although it looks very well *on paper*, has unfortunately but little foundation in nature; still it is *scientific*, the critic informs us, and consequently it is far preferable to the dull doctrines of *fact*. Every practitioner of observation, indeed, well knows that *excessive and inordinate* secretion from the skin will lessen the watery secretions from the kidnies and mucous membrane of the intestines, and that in dysentery and diabetes the perspiration is diminished. This, the critic having learned from books, he draws the sweeping conclusion of a *reverse* sympathy between the surface and internal organs, as always in operation! Yet those *unscientific* observers who happen to have studied at the bedside of sickness, as well as in the library, know that there is not a more certain means of *increasing* the secretions of the glandular organs of the abdomen, than by *increasing* the functions of the skin, keeping within the range of excess above alluded to. This is a matter of fact so well known to every practical man, that we shall not go into any farther arguments with a critic who has plainly proved himself to be totally unacquainted with the subject on which he has written with so much confidence.

But to return. Both the warm and cold bath may be usefully employed in rousing the functions of the skin, and through it the functions of the abdominal viscera, in cases of habitual constipation. The bath, impregnated with mineral acids, is also more powerful than the plain bath. Of this we are assured by personal observation; and we know that some of the first—and the *very first* physician of the age we live in, are of a similar opinion. Let those who, through prejudice, decline making the trial of this valuable auxiliary, take the consequence, in the loss of their patients, and the enhancement of their rivals' reputation. This last argument, the *ultima ratio medicorum,* will have more effect, by the bye, than any thing we can say in behalf of a useful auxiliary, which would often save the patient a great deal of mercurial and other rough remedies, by which the tone of their digestive organs is frequently injured.

Early rising, and walking before breakfast, we have found useful in constipation of the bowels; and so is a large glass of cold or warm water taken immediately on getting out of bed. The use of flannel, in hot weather, by which too much perspiration is carried off by the skin, has a tendency to constipate the bowels; but we have observed that the constipation from *this* cause is rarely productive of any bad consequences. It is where the skin and glandular organs are all torpid together that mischief most readily takes place.

Of all remedies perhaps for constitutional constipation, injections are the most effectual, *because* the most easily employed. The habit of using enemata, in this country, is rapidly increasing; and it is one of the best habits we have imported from our continental neighbours. Tepid water, with a little soap, we have found the best injection. It empties the rectum and lower portion of the colon, without any stimulus, which might ultimately render the gut insensible to the operation of glysters; while the action of the rectum appears to be communicated to the upper portions of gut, and thus a general progression of the fæcal remains effected.

In respect to medicines, we have found small doses of the quicksilver pill at bed-time, with Epsom salts, or magnesia and rhubarb in the morning, the most generally useful laxatives. But the whole class of eccoprotics must be occasionally had recourse to; especially where the auxiliary power of the nitro-muriatic acid bath is rejected through the prejudice of patient or practitioner.

In closing this article, we must request Mr. Howship to accept our best thanks for the information which the perusal of his work has afforded us, respecting several important and harassing complaints. We have no doubt but the profession generally will also profit by the experience and accurate observation of our author.

