# Errata

**Published:** 1820-09-01

**Authors:** 


					2'J8, line frorri top 15% for is} 3 read <( are.

				

